# Antiseptic Surgery1Read before the California State Veterinary Medical Association, March 13, 1895.

**Published:** 1895-05

**Authors:** H. A. Spencer


### ANTISEPTIC SURGERY.[1]

BY H. A. SPENCER, SR., V. S.

Mr. President and Gentlemen: For several years I have been deeply interested in trying to fathom the mystery of the most humane and expeditious method of treating surgical and accidental wounds. A number of years ago our journals fairly teemed with the successful results of antiseptic surgery, but the technique was either so poorly described, or rendered unavailing by the demands for extensive and expensive paraphernalia, that I, after many crude attempts, abandoned it, with the firm conviction that it had its origin in the vagaries of some visionary theorists, and was entirely impracticable in ordinary practice; therefore, I would be content to follow the time-honored custom of our fathers, and welcome the appearance of laudable pus.

But a friendly intercourse with a number of medical gentlemen who were firm adherents to modern surgery finally led to numerous invitations, which were eagerly accepted, to attend surgical operations where the aseptic and antiseptic methods were employed in the most minute detail; and, gentlemen, the revelation was made plain to me that my former attempts had been not anti- but candidly pro-septic surgery.

That the study of bacteriology has been productive of some of the most startling discoveries as to the cause of disease is readily conceded by all students of medicine. Among the most brilliant results of these investigations none have been more gratifying to the progressive practitioner than the perfecting of a methodical and scientific manner of conducting surgical operations with a view to complete asepsis.

We are accustomed to hear operators who have gleaned some theoretical knowledge of bacteriology from the casual perusal of our literature, or from the lectures of their college professors, discourse learnedly on the dangers of wound infection and the various methods to be employed to prevent such disaster; but where we witness their attempts we find them fully as inconsistent and abortive as the crude attempts that I made in the same line in my early efforts to emulate the successes of those scientists who related their providential achievements in

[1] Read before the California State Veterinary Medical Association, March 13, 1895.

the columns of the *Review*, but forgot the importance of giving their readers a lucid description of the details that blended inseparably perfect the modus operandi.

It is patent that a surgeon who would wear the laurels of a successful operator in modern surgery should have a keen conception of the relative meaning of the terms sepsis, asepsis, and antisepsis, and a fixed determination to use the knowledge in its strictest sense in his surgical practice, for the omission of a single detail, however minute, in the performance of an aseptic or antiseptic operation will in the majority of instances be the means of introducing infectious material into the wound. It is to be also remembered that the sins of commission are frequently as fertile a production of evil as that of omission. Therefore, he must not depart from the minutia of the rules laid down for his guidance.

Aseptic is a word derived from the Greek, and is defined as not liable to putrefaction; but surgically it means something more than this, as our hands, apparel, and instruments are not liable to putrefaction, or at least are only remotely so, while, unless they have been sterilized and are kept sterile, they are not aseptic, but are mediums whereby we may convey the most disastrous infections to wounds. Therefore, by aseptic we mean to bring about a condition in which there is a complete absence of infectious or septic material, or, in other words, excluding from the seat of operation and from our hands and those of our assistants, from our instruments, sponges, dressings, or any material that we may elect to use in our operation, all micro-organisms of a pathogenic nature.

And though we may have observed with most scrupulous care all the details for complete asepsis, still it is very likely that fresh wounds may contain organisms that are either numerically too few, or are non-virulent, and hence do not give rise to infection. Indeed, no method has yet been discovered by which the skin can be rendered absolutely sterile. And there are micro-organisms in cutaneous glands that the most thorough disinfecting fails to remove, and these in a suitable soil are capable of producing inflammation and suppuration. Therefore, it is patent that we should be as thorough as possible, for if these bacteria should be of a virulent nature, and the patient of an anæmic character, the tissue, fluids and cells would not exercise the germicidal power they are ordinarily endowed with, and infection would undoubtedly occur. In short, if we have not had at least an elementary training in bacteriology, we should unswervingly follow the detailed instructions of those whose researches in that field of science have made them proficient tutors to guide and direct us.

To those who thoroughly understand the phenomena that produces suppuration in wounds and general septic conditions, they are not a source of wonderment. To them it is only remarkable that these conditions do not exist more frequently. Hence, I say, that no matter how sound an anatomist or how skilful an operator you may be, unless you practice in detail the rules of asepsis when it is possible (which, unfortunately for us of the veterinary persuasion, is not as frequent as we would wish,) you will fall out of the procession of advancement that modern surgery is making.

The terms sepsis or septic infection, as has been indicated, includes all, or nearly all, the general or local surgical infections caused by bacterial invasion. The chemical products of bacteria are more the cause of disorder than they are themselves. Several varieties of micro-organisms when they gain entrance to the general circulation multiply, and then a general blood-infection is the result, which frequently proves fatal. With or without extensive multiplication of micro-organisms in the blood, the system may be overwhelmed with bacterial poisons. This condition is known as acute septicæmia. Localization of pyogenic bacteria in the organs, especially when they have been transported by emboli, give rise to multiple abscesses, and is called pyæmia, and when both conditions co-exist it is called septic-pyæmia.

Under the head of local infections are grouped together all those accidents which befall wounds: suppuration, traumatic fever, hospital gangrene, wound-diphtheria, and erysipelas. Antisepsis: In the employment of antiseptics we use the best means that can be devised for destroying the bacteria that exist both in a wound or upon the implements that shall come in contact with a wound, and here it is proper to state that the terms disinfectant and antiseptic should not be confounded. The former relates to those agents which destroy pathogenic or putrefactive organisms, and are germicides, while antiseptics only arrest putrefaction and fermentation, but do not necessarily kill the micro-organisms. A deodorizer does away with stench, but may not have either antiseptic or disinfectant properties.

While bacteriologists have shown us that infection rarely takes place from the air, they have also demonstrated that it is most frequently brought about by contact, thus we are enabled to understand the importance of preventing the introduction of bacteria on instruments or the hands of the operator and his assistants.

The association of laboratory with operative clinical experience should continue, and we cannot forego advising the expediency of the surgeon and the bacteriologist laboring together in a field that promises so much for the instruction of both when the results of their investigations are brought into comparison.

But our enthusiasm for aseptic methods should not allow us to lose sight of the necessity of perfect mechanical modes of procedure. In the matter of technique, aseptic operations call for no inconsiderable preparation; first, the operating table should be sheltered and occupy a reasonably clean room. All instruments should be subjected to a thorough cleansing and be placed in a boiling sterilized water, after which they should be distributed in convenient trays or shallow pans which should contain a sufficient amount of carbolized hot water to keep them submerged. Several gallons of sterilized salt water should be provided for use in a large fountain syringe, preferably made of metal, to admit of its sterilization. A quantity of towels, and enough loose smocks or aprons for the operator and his assistants should be prepared by boiling in a solution of 1 to 1000 of bichloride of mercury, and afterward dried and subjected to a heat of 212° in an oven, when they may be carefully wrapped up until needed; sponges should be made, not purchased. They may be prepared by taking pieces of antiseptic gauze about six inches square and placing wads of antiseptic cotton in their centre and tying them together, thus forming a very convenient cheap absorbent. There should be an abundance of these, and after having been subjected for some time to the heat of the oven, they should be packed away in sterilized wide-mouthed fruit jars.

A large piece of rubber or oil-cloth should be sterilized to be used between the field of operation and the table. The patient should be prepared for the operator by an assistant, who should see that the parts immediately adjacent to the seat of operation are shaved and thoroughly scrubbed with, first, clean hot water, soap, and brush, and then thoroughly rinsed by some antiseptic fluid from the fountain syringe, after which this part must be left untouched except with sterilized hands. The patient may be now placed upon the table and anæsthetized while the operator and assistants are preparing their hands. This should be done in the following manner: First, a thorough scrubbing with hot water and soap, then a rinse in a carbolic solution, then in a strong solution of permanganate of potash, then in a solution of oxalic acid, and a final douche from the fountain syringe, after which they may be dried by wiping on one of the sterilized towels.

From then on those concerned must be impressed with the fact that they are to touch absolutely nothing that is unsterilized; wiping off perspiration, blowing the nose, scratching the face, or putting hands in the pocket must be strictly forbidden.

The assistants are then each assigned their individual duty—one to manage the nozzle of the fountain syringe, one to hand sponges and instruments, one to sterilize instruments that have been used and are dirty, and one to see that the sterilized towels completely surround the field of operation, that the operator's hands or instruments may not come in contact with non-sterilized objects. The instruments, dressings, sponges, basin for rinsing hands, fountain syringe, etc., being now placed in convenient situations by some of the assistants, the surgeon and his assistants should be helped into their smocks and the operation may be proceeded with. And, gentlemen, if you will follow these details and finally cleanse your wound of all clots and débris, and then make your wound impervious to the air with a thoroughly antiseptic dressing, you will have an aseptic wound, and it will never require but the one dressing, for it will heal without stench, swelling, or suppuration, and there will never be an appreciable elevation of temperature.

---

Rubbers for dogs are the newest products of civilization. Salted tracks on our streets give rise to sore feet in dogs, hence this new invention. Dogs in Arctic regions used for pulling sleds have leather toe-pieces fastened with thongs.

The bill to again extend the time of registration of veterinarians for a period of six months in New York State has been quashed in the Senate through the efforts of the Legislative Committee of the New York State Veterinary Medical Association. We sincerely trust that this is the last time they will have occasion to block such a movement.

